# Efficacy of Cyclooctadepsipeptides and Aminophenylamidines against Larval, Immature and Mature Adult Stages of a Parasitologically Characterized Trichurosis Model in Mice

**DOI:** 10.1371/journal.pntd.0002698

**Published:** 2014-02-20

**Authors:** Daniel Kulke, Jürgen Krücken, Achim Harder, Georg von Samson-Himmelstjerna

**Affiliations:** 1 Institute of Parasitology and Tropical Veterinary Medicine, Freie Universität Berlin, Berlin, Germany; 2 Global Drug Discovery – Animal Health – Parasiticides, Bayer HealthCare, Leverkusen, Germany; 3 WE Biology, Heinrich-Heine-Universität Düsseldorf, Düsseldorf, Germany; Michigan State University, United States of America

## Abstract

**Background:**

The genus *Trichuris* includes parasites of major relevance in veterinary and human medicine. Despite serious economic losses and enormous impact on public health, treatment options against whipworms are very limited. Additionally, there is an obvious lack of appropriately characterized experimental infection models. Therefore, a detailed parasitological characterization of a *Trichuris muris* isolate was performed in C57BL/10 mice. Subsequently, the *in vivo* efficacies of the aminophenylamidines amidantel, deacylated amidantel (dAMD) and tribendimidine as well as the cyclooctadepsipeptides emodepside and in particular PF1022A were analyzed. This was performed using various administration routes and treatment schemes targeting histotropic and further developed larval as well as immature and mature adult stages.

**Methodology/Principal Findings:**

Duration of prepatent period, time-dependent localization of larvae during period of prepatency as well as the duration of patency of the infection were determined before drugs were tested in the characterized trichurosis model. Amidantel showed no effect against mature adult *T. muris*. Tribendimidine showed significantly higher potency than dAMD after oral treatments (ED_50_ values of 6.5 vs. 15.1 mg/kg). However, the opposite was found for intraperitoneal treatments (ED_50_ values of 15.3 vs. 8.3 mg/kg). When emodepside and PF1022A were compared, the latter was significantly less effective against mature adults following intraperitoneal (ED_50_ values of 6.1 vs. 55.7 mg/kg) or subcutaneous (ED_50_ values of 15.2 vs. 225.7 mg/kg) administration. Only minimal differences were observed following oral administration (ED_50_ values of 2.7 vs. 5.2 mg/kg). Triple and most single oral doses with moderate to high dosages of PF1022A showed complete efficacy against histotropic second stage larvae (3×100 mg/kg or 1×250 mg/kg), further developed larvae (3×10 mg/kg or 1×100 mg/kg) and immature adults (3×10 mg/kg or 1×100 mg/kg). Histotropic first stage larvae were only eliminated after three doses of PF1022A (3×100 mg/kg) but not after a single dose.

**Conclusions/Significance:**

These results indicate that the cyclooctadepsipeptides are a drug class with promising candidates for further evaluation for the treatment of trichurosis of humans and livestock animals in single dose regimens.

## Author Summary

Treatment options against whipworm infections of humans and livestock are very limited and even anthelmintics recently introduced into the market do not significantly improve the situation. Here, we evaluated members of two relatively new drug classes, the aminophenylamidines (amidantel, deacylated amidantel, tribendimidine) and the cyclooctadepsipeptides (PF1022A, emodepside) in a murine trichurosis model. While tribendimidine is licensed for the treatment of human helminthosis caused by hookworms, pinworms and roundworms in China, emodepside is the nematocidal component of dewormers for cats and dogs. With the exception of amidantel, all drugs showed good efficacies against adult whipworms using three consecutive doses. Due to considerations regarding drug safety and price, PF1022A was further evaluated against histotropic first and second stage larvae, further developed larvae, immature and mature adults using a single or three consecutive doses. Three doses eliminated all stages while a single dose was inefficient against histotropic first stage larvae. In general, higher doses were required for early stages in comparison to stages protruding into the gut lumen. Since only a very basic formulation of drugs was tested, further improvement can be expected from optimized formulations. Cyclooctadepsipeptides should therefore be considered as candidates for evaluation to treat *Trichuris* spp. infections in livestock and humans.

## Introduction

About 20 major human helminthoses have a significant impact on global public health [1]. Since a highly disproportionate share of the burden occurs in developing areas of sub-Saharan Africa, Asia and the Americas, helminth infections belong to both, the “neglected tropical diseases” and the “neglected infections of poverty” [2], [3]. In these regions more than a billion people are infected with one or more worm species [2]. An important part of human helminth infections worldwide is caused by soil-transmitted nematodes, including the roundworm *Ascaris lumbricoides* with 800 million infections, the whipworm *Trichuris trichiura* with 600 million infections, and the hookworms *Ancylostoma duodenale* and *Necator americanus* with 600 million infections [4]. An estimated 1.6–6.4 million disability adjusted loss of life years are a direct result of trichurosis [4]. In 2010 an estimated 5023 million people lived in areas stable for transmission of *Trichuris trichiura*, plus another 284 million lived in areas of unstable transmission of whipworms, globally [5]. High prevalence often comes along with high abundance of protein energy malnutrition and anemia as well as limited access to medical care and educational opportunities [6]. Mild *T. trichiura* infections are often asymptomatic, but severe and chronic infections can result in the *Trichuris* dysentery syndrome including chronic inflammation of the intestine, rectal prolapse, anemia, poor growth, and clubbing of the fingers [6].

Despite the strong impact of helminthoses on public health, only four anthelmintics (albendazole, mebendazole, levamisole, and pyrantel) with only two different modes of action are listed on the WHO list of essential medicines to treat soil-transmitted nematode infections [7] with mebendazole and albendazole being by far the most commonly used drugs [8]. Whereas both drugs are highly effective against adult *A. lumbricoides* in a single dose, only albendazole is used for the treatment against tissue migrating larvae – mebendazole is poorly absorbed from the gastrointestinal tract thus its therapeutic activity is largely confined to adult/luminal worms [8]. Furthermore, the efficacy of both drugs is unsatisfactory against hookworms and *T. trichiura* in single dose regimen [9]. Higher efficacies against whipworms and hookworms were observed when albendazole or mebendazole were administered using multiple drug administration [10]. However, treatments using multiple doses significantly increase costs and management efforts in particular in poor communities lacking efficient public health infrastructure. Moreover, persistent underdosing of *A. duodenale*, *N. americanus* and *T. trichiura* within recently increased large-scaled mass drug administration campaigns against filariosis and soil-transmitted helminthosis may favor selection of highly resistant genotypes [9] as already described for *T. trichiura* [11].

In addition to its relevance in human medicine, the genus *Trichuris* also has an enormous impact on veterinary medicine. For instance, *Trichuris vulpis*, the dog whipworm, causes an intestinal parasitosis of clinical relevance and is also suspected to be zoonotic [12]. However, several anthelmintics registered for use in dogs such as diethylcarbamazine, piperazine, ivermectin and pyrantel lack efficacy against *T. vulpis* severely limiting the choice of drug for deworming [12]. In swine, infections with *Trichuris suis*, the dose-limiting nematode for all relevant anthelmintic drug classes, lead to reduced growth rates and therefore result in significant economic losses [13]. Finally, due to the long period of prepatency of *Trichuris* spp. and the lack of efficacy of most drugs against histotropic larval forms, two blocks with one to three doses each are usually necessary to completely eliminate the parasites [12].

It is therefore obvious, that the development of new, safe and highly efficacious drugs to treat soil-transmitted nematode infections is urgently required. In particular, new drugs for the treatment of *Trichuris* spp. using a single dose would significantly increase treatment options in both, human and veterinary medicine. Therefore, the evaluation of the efficacy of promising drug candidates against whipworms is an essential step towards improvement of anthelmintic treatment opportunities.

To investigate and compare the anthelmintic profiles of new drug candidates against whipworm infections, the *Trichuris muris* mouse model is highly suitable [14]. *Trichuris* L1 hatch in the small intestine of their host and migrate rapidly to the caecum and colon [15], where they invade the epithelium [16] and undergo a histotropic phase with two molts lasting several days (duration depends on the particular species and isolate). Then, larvae migrate to the surface of the epithelium extruding their caudal ends freely into the lumen of the intestine (further developed larvae or free larvae) [16]. In general anthelmintics have been reported to be less effective against histotropic larvae, which might be attributed to the poor accessibility of drugs to these larvae within the tissue [12].

In order to eliminate parasites using a single dose or at least a single treatment block, it is desirable to evaluate drug candidates not only against mature adult worms but also against histotropic larvae and further developed immature stages. Since duration of development and timespan of infection depend on both, the host strain [17] and whipworm isolate [18], a detailed characterization of the respective host-parasite relationship is essential. Thus, localization of larvae in the course of the prepatent period and onset of patency of the infection have to be analyzed carefully before *in vivo* assays against specific stages of *T. muris* can be conducted meaningfully with the respective isolate.

The cyclooctadepsipeptides [19] and the aminophenylamidines [20] are promising anthelmintic classes for further development of broad-spectrum drugs to treat intestinal nematode infections. The semi-synthetic cyclooctadepsipeptide emodepside has been shown to have an almost complete efficacy against immature and mature stages of *T. vulpis* in dogs [21] and *T. muris* in mice [22], [23] while the aminophenylamidines amidantel and tribendimidine showed only low to moderate efficacy against *T. muris* in mice [24] and *T. trichiura* in humans [25]–[27].

Both drug classes have completely different target molecules. It is clear that the aminophenylamidines are agonists of acetylcholine receptors and have a very similar mode of action as levamisole [28], [29] whereas several targets have been suggested for the cyclooctadepsipeptides with the voltage-gated, calcium-activated potassium channel SLO-1 as most important candidate [19], [30], [31]. However, the G-protein coupled receptor LAT-1 [32] and ionotropic GABA_A_ receptors [33], [34] might also contribute to susceptibility to cyclooctadepsipeptides.

Therefore, the present study investigated and compared the *in vivo* anthelmintic properties of the semi-synthetic cyclooctadepsipeptide emodepside, its parental natural fermentation product PF1022A and the aminophenylamidines amidantel, deacylated amidantel and tribendimidine against *T. muris*. Since tribendimidine has previously been reported to have insufficient activity after oral administration in humans [25]–[27], drugs were also administered intraperitoneally and subcutaneously. In addition to the evaluation of adulticidal efficacy, PF1022A was further tested against histotropic larvae and further developed immature stages of whipworms, using single and three consecutive doses.

## Materials and Methods

### 2.1 Ethical statement

All studies presented were conducted at the laboratories of Bayer HealthCare, Global Drug Discovery, Animal Health in Monheim, Germany. The experiments were registered and approved by the State Office for Nature, Environment, Agriculture, and Consumer Protection, North Rhine-Westphalia, Germany (reference number 200/V14), in accordance with §8a, Section 1 and 2 of the German Protection of Animals Act and the European Union directive 2010/63/EU.

### 2.2 Drugs

Amidantel, dAMD, emodepside and PF1022A were available at Bayer HealthCare AG, Global Drug Discovery Animal Health in Monheim, Germany. Tribendimidine was obtained from Shandong Xinhua Pharmaceutical Company Limited (Zibo, People's Republic of China). All drugs were stored at 4°C until further use. Individual drug concentrations were prepared separately as dispersions in Cremophor EL (BASF, Ludwigshafen, Germany) and deionized-water [1∶3] on the days of treatment.

### 2.3 Animals and parasites

Female SPF inbred mice of the strain C57BL/10 ScSnOlaHsd (C57BL/10) were purchased from Harlan UK Limited, at four weeks of age. They were housed in Macrolon cages under environmentally controlled conditions and kept in groups of five animals unless otherwise indicated. Water and Sniff rodent food pellets were available *ad libitum*. Mice were allowed to acclimate for exactly seven days before starting any experiments. The *T. muris* isolate was kindly provided by Heinz Mehlhorn (Düsseldorf, Germany). A detailed history regarding isolation and passage is not available.

Mice were orally infected with a gavage using 0.2 ml fresh tap water with 200 eggs containing fully developed L1 of *T. muris*. Murine feces were collected on days 49, 56 and 63 p.i., euthanasia was performed by carbon dioxide suffocation.

Isolation of the eggs was performed as described in section 2.4.1. The development of L1 in the eggs was performed in stender dishes in an incubator at 27°C and 95% humidity for approximately 8 weeks. Progress of embryonation was controlled weekly. After development of L1 in >90% of the eggs was completed, eggs were stored at 4°C until further usage for a maximum of 6 months. Before infection of mice, the egg suspension was washed with fresh tap water at room temperature.

### 2.4 Parasitological characterization of a *T. muris* life cycle in C57BL/10 mice

#### 2.4.1 Determination of the periods of prepatency and patency

To assess the duration of prepatent period, ten mice were infected. Starting from day 7 p.i., all ten mice were housed on grids to collect feces for 24 h once a week. During these periods, the bottom of the cage was covered with 300 ml tap water. Feces and water were collected in a 1 l beaker and homogenized with a hand-held blender. Using a wooden spatula, fine components of the feces were separated from remaining debris by filtration through a 200 µm sieve and collected in a clean 1 l beaker. The residues were rinsed with tap water until the filtrate reached a volume of 600 ml. After sedimentation for 1 h, the supernatants (approximately 500 ml) were removed. The sediment was centrifuged at 2,000×g and room temperature for 10 min. The pellet was resuspended in 200 ml tap water and centrifuged under the same conditions. After another washing step, the pellet was resuspended in 200 ml saturated sodium chloride solution. Then, samples were centrifuged at 2,000×g and room temperature for 5 min, the top 25 ml were filled into a 300-ml beaker and 225 ml tap water were added. After at least 2 h of sedimentation the supernatant was decanted and the sediment was washed in tap water another four times. After decanting the supernatant, the sediment (approximately 20 ml) was examined for the presence of eggs. Examination of feces was continued until three consecutive samples were found to be negative. Three independent experiments with ten mice each were performed.

#### 2.4.2 Variation in egg output in the course of patency of the infection

To determine the variation in egg output in the course of patency of the infection, a fecal egg count method was adapted from Stoll [35]. In brief, 10 mice were infected. Only animals, positive for eggs in their feces on day 35 p.i., were included in the study. Starting from day 35 p.i., mice were housed individually on grids in Macrolon cages to collect individual feces for 12 h periods once a week. Fecal samples (0.5 g) were weighed from each mouse, 7.0 ml water were added and incubated for 15 min. Feces were roughly macerated with a wooden spatula followed by an extensive homogenization using a magnetic stirrer at low speed until samples were analyzed. For each sample, three 75 µl aliquots were pipetted on microscope slides and eggs were counted. To obtain the number of eggs per gram feces, the arithmetic mean of the three counts was multiplied by 200 to calculate the number of eggs per gram feces (epg). Feces were analyzed until 15 weeks p.i., since status of patency of the infection became quite variable afterwards (see 2.4.1 and 3.1.1).

#### 2.4.3 Time course of localization of larvae in the course of prepatent period

To analyze the time course of the localization of larvae during prepatent period the following experiment was adapted from Panesar [36]. For this experiment 120 mice were infected. During the first 40 days p.i., three mice were euthanized daily and their duodena, caeca and colons were removed and split open. The luminal content was removed and inspected for any stages of *T. muris*. Then, the mucosa of the guts was examined for the presence of worms extruding into the lumen of the guts. Finally, duodena, caeca and colons were cut into small squares and separately incubated in 0.85% physiological sodium chloride solution at 37°C for 24 h. By carefully scraping the mucosa the histotropic larvae became visible using a dissecting microscope. Seven mice, in which not a single stage of *T. muris* was found, were excluded from the study.

#### 2.4.4 Female/male ratio in the course of infection

On day 35 p.i., fecal examinations were performed for each of the 60 infected mice individually to confirm patency of the infection. Only animals found positive for eggs in their feces were included in the study. Weekly, starting from day 35 until day 152 p.i., three mice were euthanized and dissected. Female and male whipworms in caecum and colon were counted. Two independent experiments with 60 mice in each experiment were performed.

#### 2.4.5 *In vitro* embryonation of *T. muris* eggs

The embryonation of eggs was analyzed and compared under several different conditions. Freshly isolated and purified eggs were suspended in (i) 0.5% formaldehyde in physiological sodium chloride solution, (ii) physiological sodium chloride solution or (iii) tap water and transferred into 40 ml stender dishes (see [37]) to compare the rate and speed of development. The progress of embryonation was assessed weekly by microscopic analysis of three 10 µl aliquots. Eggs were counted and categorized as (i) unembryonated, (ii) partially embryonated, (iii) fully embryonated or (iv) degraded. The latter category was chosen according to the following criteria: a) vesicular appearance of unsegmented eggs or b) deformed larval structures within the eggs.

Furthermore, incubation temperatures of 4°C, 19°C, 27°C and 37°C as well as the influence of the presence of antibiotic (i.e. 10 µg/ml sisomycin plus 1 µg/ml clotrimazole), relative humidity (75%, 85% and 95%) and light conditions (light versus no light) were evaluated in tap water using the same method.

Finally, the influence of storage at 4°C after full embryonation of eggs was compared to storage at 27°C to determine the best storage condition. After embryonation at 27°C, eggs were stored at 27°C or at 4°C for 70 days. For each incubation temperature, 5 mice were infected. On day 45 p.i., mice were euthanized and worm counts were determined.

### 2.5 *In vivo* efficacy against *T. muris* in mice

In 24 consecutive experimental blocks, 655 mice were randomized into 132 groups, each consisting of five animals. One group of 5 mice was used for each dosage and for each administration route tested. In each block, 5 infected mice served as untreated control and received the vehicle only.

#### 2.5.1 *In vivo* efficacy against mature adult stages of *T. muris*

On day 42 p.i., a fecal examination was performed for each mouse to confirm patency of the infection. Only animals positive for *T. muris* eggs in their feces were included in the study. Based on the individual body weight on day 45 p.i., exact dosages were calculated. In case of multiple dose regimens, three doses of the respective drug were administered orally, intraperitoneally or subcutaneously (nuchal fold) on days 46–48 p.i. Dosages used are summarized in Table 1. For single doses, 50, 75, 100, 150, 200, 250, 300 or 500 mg/kg PF1022A were administered on day 48 p.i. On day 49 p.i., mice were euthanized. Subsequently, necropsy was performed and worms in colons and caeca were counted.

**Table 1:** Single and multiple drug dosages evaluated *in vivo* against mature adults of *T. muris*, classified by route of administration.

Dosage (mg/kg)		0.5	1.0	2.5	5.0	7.5	10	15	20	25	50	75	100	150	200	250	300	400	500
**PF1022A**	**3**×**oral**		X	X	X	X	X			X	X		X						
	**1**× **oral**										X	X	X	X	X	X	X		X
	**3**× **subcutaneous**										X		X	X	X	X	X	X	X
	**3**× **intraperitoneal**						X			X	X	X	X	X	X	X			
**Emodepside**	**3**× **oral**	X	X	X	X	X	X				X		X						
	**3**× **subcutaneous**		X				X	X	X	X	X	X	X						
	**3**× **intraperitoneal**		X		X		X	X	X	X	X		X						
**Amidantel**	**3**× **oral**																		X
	**3**× **subcutaneous**																		
	**3**× **intraperitoneal**																		
**dAMD**	**3**× **oral**		X		X		X			X	X		X						
	**3**× **subcutaneous**												X						X
	**3**× **intraperitoneal**				X		X			X	X		X						
**Tribendimidine**	**3**× **oral**		X		X		X			X	X		X						
	**3**× **subcutaneous**												X						X
	**3**× **intraperitoneal**				X		X			X	X		X						

‘X’ indicated that the respective drug was evaluated in the given dose using the indicated route of administration.

#### 2.5.2 *In vivo* efficacy against larval and immature adult stages of *T. muris*

Based on the parasitological characterization (duration of prepatent period and time-dependent localization of larvae during period of prepatency), the *in vivo* efficacy of PF1022A was also investigated against larval and immature adult stages of *T. muris*. According to the time course of localization of developmental stages in the present study and the analysis on the molting pattern in *T. muris* [36], the following time points for drug administration were chosen:

Individual body weight was determined on the day of infection for L1, on day 11 p.i. for L2 and on day 25 p.i. for further developed stages. PF1022A dosages of 10, 100, 250, and 500 mg/kg or 1.0, 10, 50 and 100 mg/kg were administered on day 3 p.i. or on days 1–3 p.i. to target L1. For the evaluation of efficacy against the histotropic L2, treatments were carried out with PF1022A dosages of 10, 100, 250, and 500 mg/kg or 1.0, 10, 50 and 100 mg/kg on day 14 p.i. or on days 12–14 p.i., respectively. Since the following molts of males and females are less synchronous [36], treatments were directed against further developed immature stages in general. Treatments with 1.0, 10, 50 and 100 mg/kg PF1022A were performed on three consecutive days (26–28 p.i.) and treatments with 10, 100, 250, and 500 mg/kg PF1022A only on day 28 p.i. Independently of the targeted stage euthanasia of mice and worm counts were performed on day 49 p.i.

### 2.6 Calculation of dose-response curves and statistical analysis

For parasitological characterization of the *T. muris* isolate used in C57BL/10 mice, descriptive statistics were performed using GraphPad Prism 5.03. Differences in worm counts between different weeks of infection and in sex ratio were determined by a One Way ANOVA followed by Dunnet's post hoc test using the first week of the patent period as the control against which all other time points were tested.

For all drugs and routes of administration tested against patent *T. muris* infections, the reduction of the worm burden expressed in percent of the corresponding control groups of 5 mice was plotted against the log_10_ of the drug dosages. Efficacies were set to zero if mean of the worm counts was higher than the mean of the corresponding control group. Furthermore, the corresponding SEM values of the affected groups start from 0 (all figures showing dose- response curves). Four-parameter-logistic curves were fitted using GraphPad Prism 5.03 [38]. The top was constrained to values between 0 and 100%. The no-drug controls were set to 10^−4^ mg/kg to allow log_10_ transformation of dosages. Calculated ED_50_ and ED_95_ values were compared using the extra sum of squares F test. If multiple tests were performed, p values were corrected using the Bonferroni-Holmes procedure.

The absolute number of recovered mature adult worms after treatment against larval and immature adult stages was compared to the number of worms isolated from the negative controls by using the non-parametric Kruskal-Wallis test with Dunn's *post hoc* test for identification of significant differences between individual groups.

## Results

### 3.1 Time course of *T. muris* development in C57BL/10 mice

#### 3.1.1 Periods of prepatency and patency of the infection

In each of the three independent experiments determining presence of eggs in feces in weekly intervals, first eggs were found on day 35 p.i. Therefore, prepatent period lasted for at least four but not longer than five weeks. None of the mice that became patent stopped shedding eggs before week 14 p.i. However, starting from week 15 p.i., samples were much more heterogeneous. Mice in experiment 1 remained patent at least until week 16 p.i., whereas animals in experiment 2 stopped shedding eggs in week 15 p.i. In experiment 3, fecal examination was negative in week 16 p.i., but was positive in week 17 p.i., again. Patency of the infection ended in week 18 p.i.

#### 3.1.2 Detailed analysis on egg shedding

**Figure 1:**
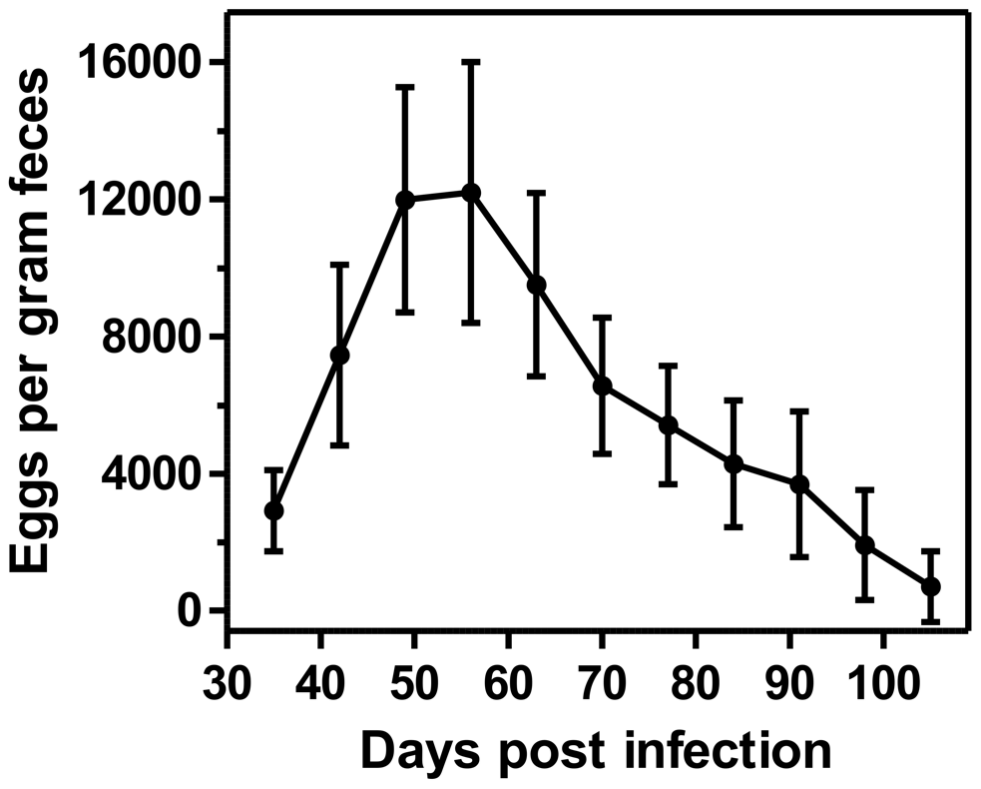
Analysis on egg shedding in the course of patency of the infection. The graph shows the arithmetic mean values with standard deviations of the absolute numbers of eggs per gram feces between days 35 and 105 p.i. with a group size of nine animals. Due to the death of one mouse, group size was reduced to eight starting from day 70 p.i.

Nine out of ten mice were found positive for eggs in their feces on day 35 p.i. Therefore, only a single mouse was excluded from the study. Furthermore, one individual mouse died on day 67 p.i. and was only included in the statistics until day 63 p.i. Variation in egg shedding in the course of patency of the infection is summarized in Figure 1. Strong variation in individual egg counts was observed, indicated by the relatively large standard deviations (Figure 1). In general, the mean eggs per gram feces (epg) increased during the first three weeks of patency of the infection starting with a mean epg of 2919±1182 (range 800–5600) at day 35 p.i. The peak in egg output was observed in week 8 p.i. (epg = 12200±3813, range 4200–19800). Starting from week 9 p.i., a gradual reduction of the average egg count was observed. In week 14 p.i. the first mouse was found to be negative for eggs in its feces. In another two mice patency of the infection ceased in week 15 p.i. For the remaining five mice the mean (± SD) of eggs per gram feces was calculated to be 1120±1110.5 (range 200–3600) on week 15 p.i., being the final week of this analysis.

#### 3.1.3 Localization of developmental stages throughout infection

**Figure 2:**
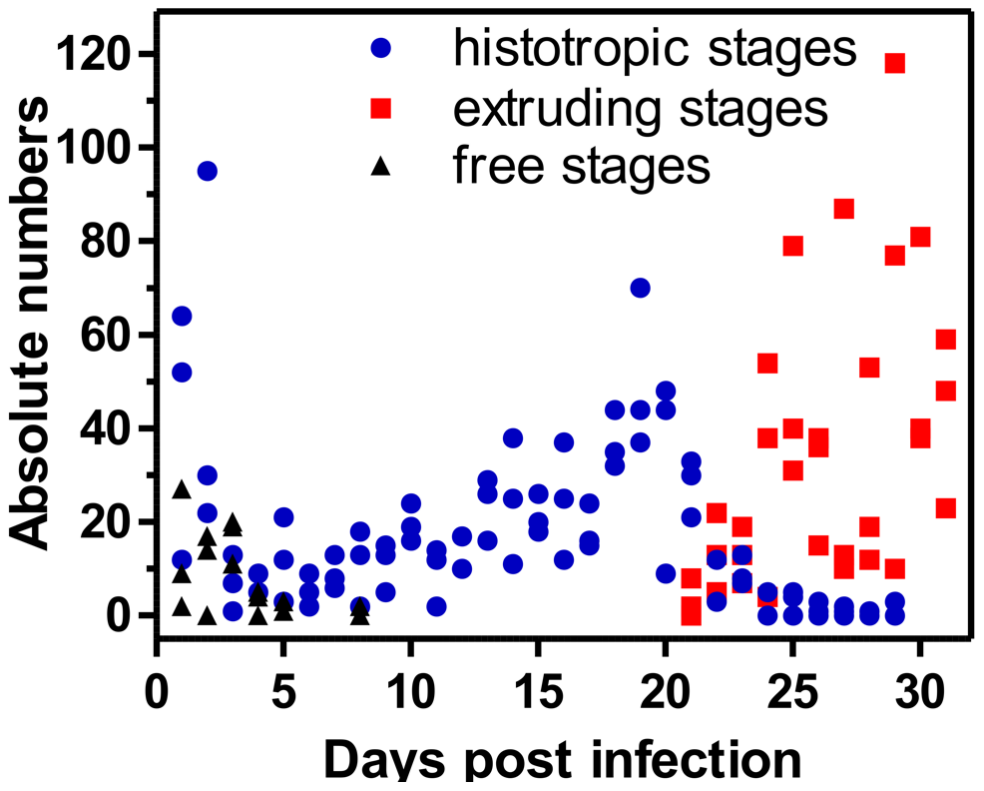
Analysis on the occurrence of specific stages of *T. muris* in the course of the period of prepatency. Presented is the occurrence of first stage larvae in the luminal content of the guts (free stages), of histotropic first, second and third stage larvae (histotropic stages) and of third and fourth stage larvae as well as immature and mature adults attached to the epithelium while extruding their posterior parts into the lumen of the guts (extruding stages) between days 1 and 31 p.i. Based on dissection of three mice per time point, the graph shows three data points for each stage and time point. If the count was found to be zero for a specific stage in each of the three independent counts, data points are not shown.

The analysis of the time course of the migration of *T. muris* stages during the period of prepatency revealed distinct phases of localization. Figure 2 summarizes the trend in absolute numbers of recovered stages in the course of the prepatent period. Supplementary Table S1 shows the individual counts divided by duodenum, caecum, colon and luminal debris.

On days 1–4 p.i. a small number (2.8±1.9, mean ± SD) of embryonated eggs was recovered from the intestinal debris of duodenum, caecum and colon. After day 5 p.i. no embryonated eggs were found in the gastrointestinal tract. Free larvae were identified in the debris of guts also for a very limited period during the first days after infection. Whereas 9.0±6.2 free larvae were recovered between day 1 and day 5 p.i. only one sample on day 8 was found positive for two free larvae. However, starting from day 27 an increasing number of immature and mature adult worms in the debris was counted (see Supplementary Table S1).

Histotropic larvae were recovered almost throughout the whole evaluation period. However, during the period of prepatency two relative maxima in histotropic larval counts were observed. A high number of histotropic larvae was detected on days 1 and 2 p.i. (45.8±4.5), while only a small number was recovered between days 3 and 12 p.i. (10.3±4.1). Starting from day 13 the number steadily increased until day 19 p.i., where 50.3±17.4 larvae were counted (see Supplementary Table S1). From day 20 p.i. on, the number of histotropic larvae decreased again and finally, starting from day 24 p.i., the majority of the guts was found to be negative. Further developed stages were not found before day 21 p.i. The number of these stages then increased until day 24 and remained stable (42.6±13.9) until the end of the evaluation period (see Figure 2). As expected, neither histotropic larvae nor any further developed stages were found in the duodenum (Supplementary Table S1). On days 30 and 31, the intestinal debris became positive for unembryonated eggs, indicating the start of patency of the infection (Supplementary Table S1).

#### 3.1.4 Worm counts and sex ratio

On day 35 p.i., 107/120 mice harbored a patent infection (infection rate of 89.17%). 13 uninfected mice and 4 mice which had died in the course of the experiment were exclude from the analysis.

**Figure 3:**
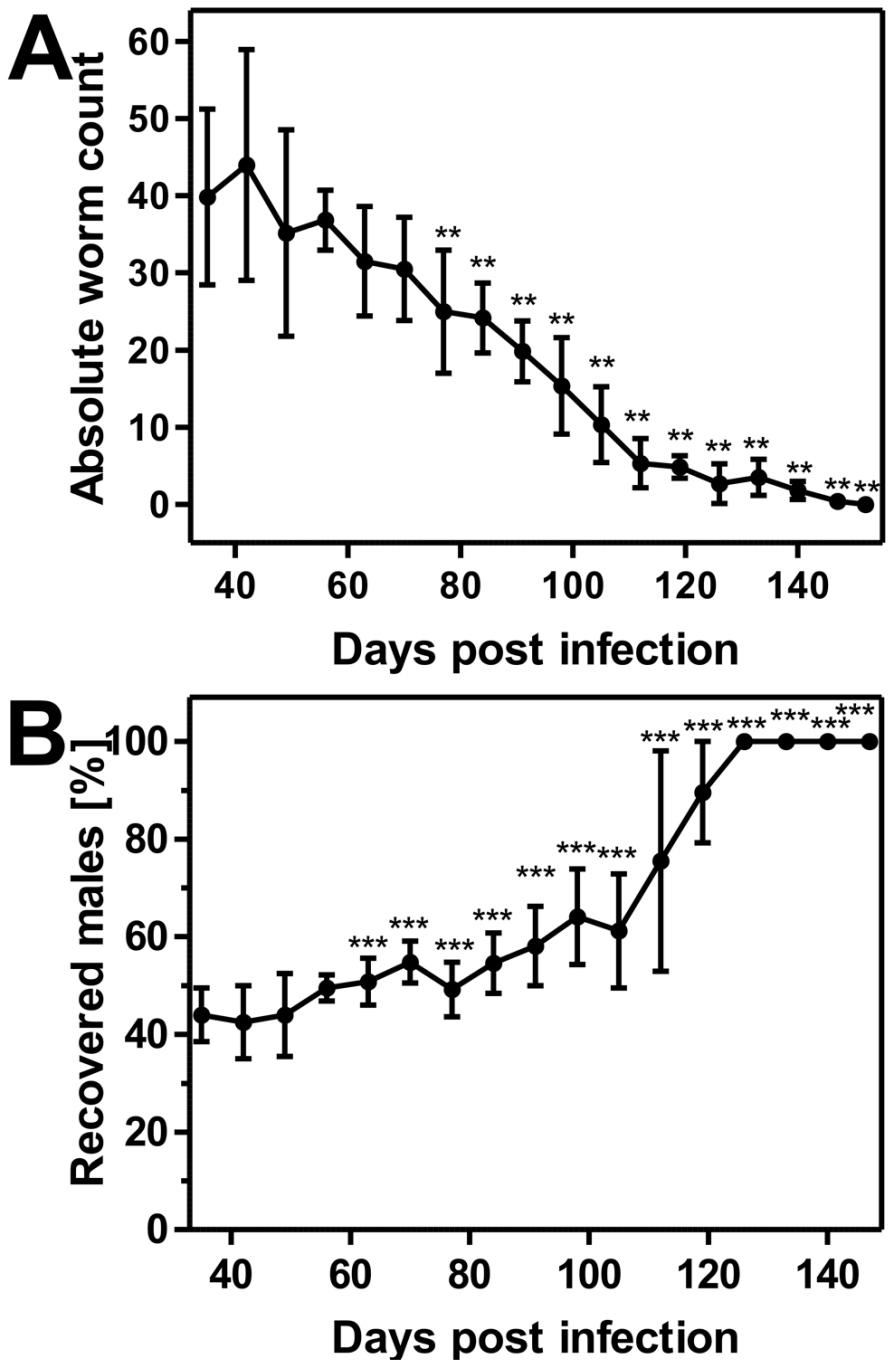
Analysis on the occurrence of *T. muris* in the course of patency of the infection. (A) Absolute worm counts in the course of patency of the infection. The graph shows the arithmetic mean values and standard deviations of the absolute number of recovered worms during time with a group size of six animals per time point. Mean worm counts were compared to day 35 p.i. using One-Way-ANOVA followed by Dunnet's post hoc test. **, p<0.01 vs. day 35. (B) Sex ratio of *T. muris* in the course of patency of the infection. Graph shows the arithmetic means with standard deviations of the recovered male worms expressed as percentage of total recovered worms with a group size of six animals per time point. ***, p<0.001 vs. day 35.

The absolute worm counts per infected host are summarized in Figure 3A. Mean worm counts were not significantly different from those on day 35 p.i. up to day 70 p.i. (One Way ANOVA followed by Dunnet's post hoc test, p>0.05) although a tendency to lower and steadily decreasing mean worm counts was observed already at earlier time-points. Thereafter, mean worm counts were significantly lower than on day 35 (p<0.01) and a continuous decrease in recovered worms was observed (Figure 3A). On day 112 p.i. only five worms per mouse where recovered on average and finally on days 145 and 152 p.i. only two whipworms were found in one of the necropsied mice. In addition to the absolute worm counts, Figure 3B shows the relative sex distribution of the worms during the same evaluation period. The male/female ratio was progressively skewed towards male worms. The ratio was 1∶1.27 5 weeks p.i, 1∶0.97 9 weeks p.i., 1∶0.56 14 weeks p.i., and 1∶0.12 17 weeks p.i. Starting in week 9, the male/female ratio was significantly higher than on day 35 p.i. (One Way ANOVA followed by Dunnet's post hoc test, p<0.001). From week 18 on, 100% of the recovered worms were males (see Figure 3B).

#### 3.1.5 Optimized conditions for *in vitro* embryonation of *T. muris* eggs

The influence of different media on the rate and speed of embryonation were compared. No significant difference was observed between (i) 0.5% formaldehyde in physiological sodium chloride solution, (ii) physiological sodium chloride solution and (iii) tap water (data not shown). Therefore, tap water was used as medium for the following analyses. The incubation temperature (4°C, 19°C, 27°C or 37°C) had an enormous impact on both speed and embryonation rate (Supplementary Table S2). Speed of embryonation steadily increased with temperature. However, at 37°C the absolute number of degenerated eggs was also increased. Additives such as sisomycin plus clotrimazole or lighting conditions did not influence embryonation and were therefore neglected. However, relative humidity (75%, 85% and 95%) strongly affected the loss of medium by evaporation and therefore 95% humidity was chosen for routine purposes.

Finally, the influence on storage temperature on egg infectivity after full embryonation was tested. Mice infected with eggs stored at 27°C or at 4°C for at least 70 days were necropsied on day 45 p.i. The infection levels between both groups were not found to differ significantly, as illustrated by worm counts ranging between 28 and 45 or 12 and 59 (p = 0.69 using the Mann Whitney U test).

### 3.2 *In vivo* efficacy of cyclooctadepsipeptides and aminophenylamidines against *T. muris*

The average number of worms recovered from caecum and colon from untreated control mice on day 49 was 33.77±15.59. Worm counts after treatment against developmental stages were also determined on day 49 p.i. (see 2.5.2), while four mice died before evaluation and were, therefore, not included in the statistics. The highest worm count was 80, whereas no worms were recovered in two cases.

#### 3.2.1 *In vivo* efficacy of aminophenylamidines against mature adult stages of *T. muris*

**Figure 4:**
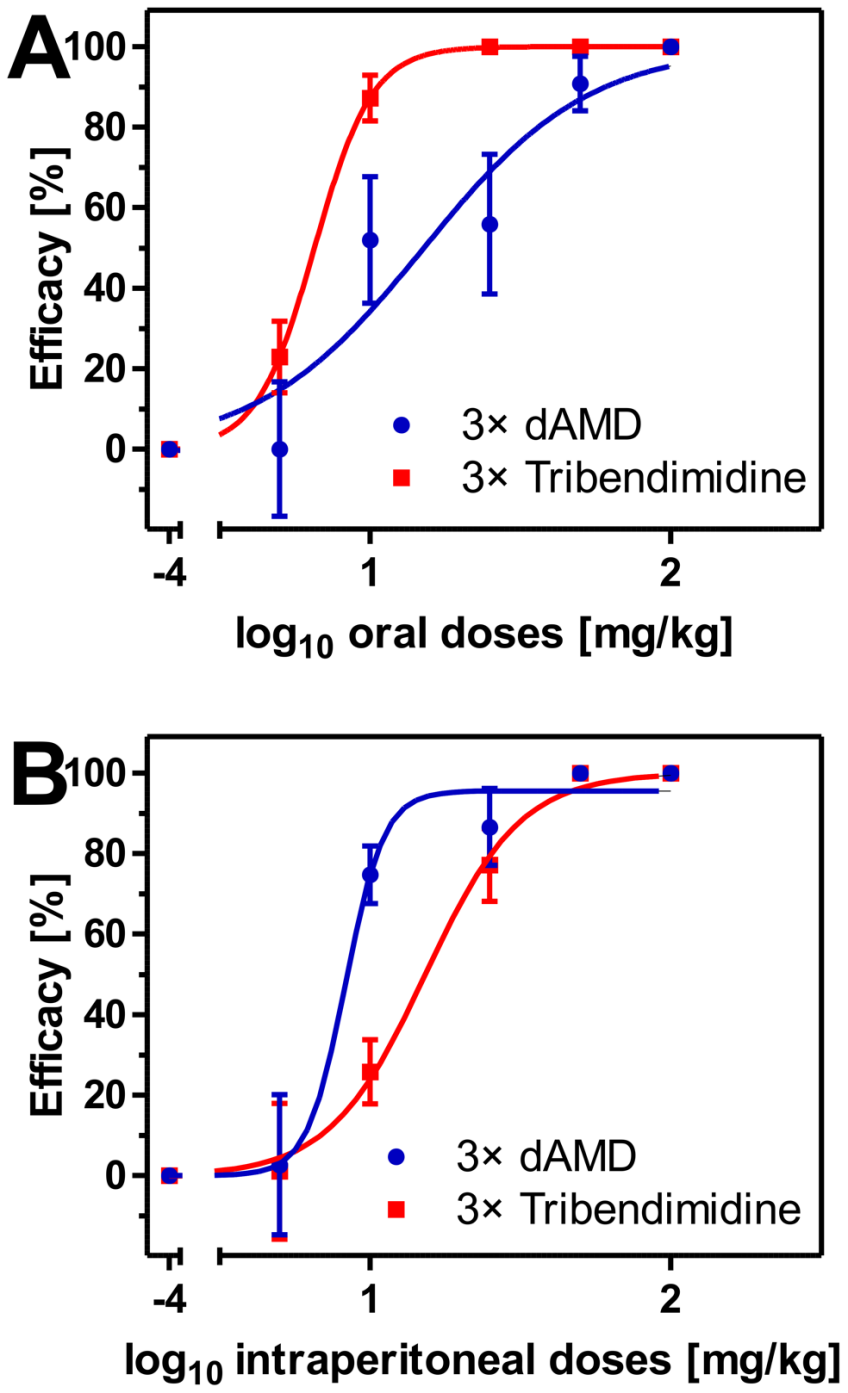
*In vivo* dose-response curves of dAMD (blue) and tribendimidine (red) after oral (A) and intraperitoneal (B) treatments against mature adults of *T. muris*. Dose-response curves show the arithmetic mean values and standard errors of the mean with a group size of five animals per drug and dose. Efficacy was calculated as relative number of recovered worms compared to the no-drug control in percentage. Dosages were log_10_ transformed and logistic regressions were calculated with top values constrained between 0 and 100%. Efficacies were set to zero if mean of the worm counts was higher than the mean of the corresponding control group. Furthermore, the corresponding SEM values of the affected groups start from zero. The no-drug controls were set to 10^−4^ mg/kg to allow log_10_ transformation of dosages.

Three oral doses of 500 mg/kg of the aminophenylamidine amidantel led to no significant reduction of the worm burden. Since three high consecutive doses of amidantel did not reduce worm counts in comparison to the no-drug control, this derivative was not further evaluated in the present study. In contrast to amidantel, both oral and intraperitoneal treatments with either tribendimidine or dAMD resulted in dose-dependent reductions of the *T. muris* burden. Dose-response curves for both drugs and both routes of administration are given in Figure 4. Furthermore, ED_50_ and ED_95_ values with 95% confidence intervals as well as p values from comparisons between the derivatives and *R^2^* values are summarized in Table 2.

**Table 2:** Comparison of the *in vivo* efficacies of the aminophenylamidines amidantel, dAMD and tribendimidine and the cyclooctadepsipeptides emodepside and PF1022A against patent *Trichuris muris* infections in mice.

Drug	Admin.	ED_50_ with 95%CI (in mg/kg)	p value^a^	ED_95_ with 95% CI (in mg/kg)	p value^b^	*R* ^2^
**dAMD**	3× oral	15.1 (9.9–22.9)	<0.0001 (vs. 3× tribendimidine oral)	97.3 (28.3–334.2)	0.0007 (vs. 3× tribendimidine oral)	0.8039
	3× i.p.	8.3 (7.3–9.5)	<0.0001 (vs. 3× tribendimidine i.p.)	12.8 (10.6–15.4)	<0.0001 (vs. 3× tribendimidine i.p.)	0.9349
**Tribendimidine**	3× oral	6.5 (6.0–7.2)	<0.0001 (vs. 3× dAMD oral)	12.6 (9.9–15.9)	0.0007 (vs. 3× dAMD oral)	0.9447
	3× i.p.	15.3 (13.2–17.7)	<0.0001 (vs. 3× dAMD i.p.)	44.8 (30.9–65.0)	<0.0001 (vs. 3× dAMD i.p.)	0.9279
**Emodepside**	3× oral	2.7 (1.9–3.9)	0.0009 (vs. 3× PF1022A oral)	24.5 (8.7–68.8)	0.3684 (vs. 3× PF1022A oral)	0.8368
	3× i.p.	6.1 (4.8–7.7)	<0.0001 (vs. 3× PF1022A i.p.)	40.0 (18.9–84.5)	<0.0001 (vs. 3× PF1022A i.p.)	0.9274
	3× s.c.	15.2 (13.0–17.7)	<0.0001 (vs. 3× PF1022A s.c.)	40.7 (24.5–67.4)	<0.0001 (vs. 3× PF1022A s.c.)	0.8481
**PF1022A**	3× oral	5.2 (4.0–6.8)	0.0009 (vs. 3× emodepside oral)	36.5 (14.9–89.8)	0.3684 (vs. 3× emodepside oral)	0.8681
	3× i.p.	55.7 (44.4–70.0)	<0.0001 (vs. 3× emodepside i.p.)	208.5 (99.2–438.2)	<0.0001 (vs. 3× emodepside i.p.)	0.8657
	3× s.c.	225.7 (180.2–282.6)	<0.0001 (vs. 3× emodepside s.c.)	515.0 (254.8–1041)	<0.0001 (vs. 3× emodepside s.c.)	0.7432
	1× oral	186.6 (111.0–313.5)	<0.0001 (vs. 3× PF1022A oral)	686.7 (168.5–2798)	<0.0001 (vs. 3× PF1022A oral)	0.6086

Presented are the ED_50_ and ED_95_ values with 95% confidence intervals (CI) and coefficients of determination (*R* ^2^) as well as p values, for determination of significant differences.

a Significant difference in ED_50_ to drug in brackets.

b Significant difference in ED_95_ to drug in brackets.

The ED_95_ of tribendimidine was found to be approximately eight times lower than the ED_95_ of dAMD following three oral consecutive doses, whereas the ED_95_ of tribendimidine was approximately four times higher than the ED_95_ of dAMD after three intraperitoneal administrations (Table 2). However, three subcutaneous doses with 100 mg/kg or 500 mg/kg of either tribendimidine or dAMD had no effect on worm counts in comparison to the vehicle treated group (data not shown).

#### 3.2.2 *In vivo* efficacy of cyclooctadepsipeptides against mature adult stages of *T. muris*

Three oral, intraperitoneal or subcutaneous doses of either emodepside or PF1022A on days 46–48 p.i., resulted in dose-dependent reductions of the *T. muris* burden. Table 2 summarizes ED_50_ and ED_95_ values as well as comparisons between them by administration route.

**Figure 5:**
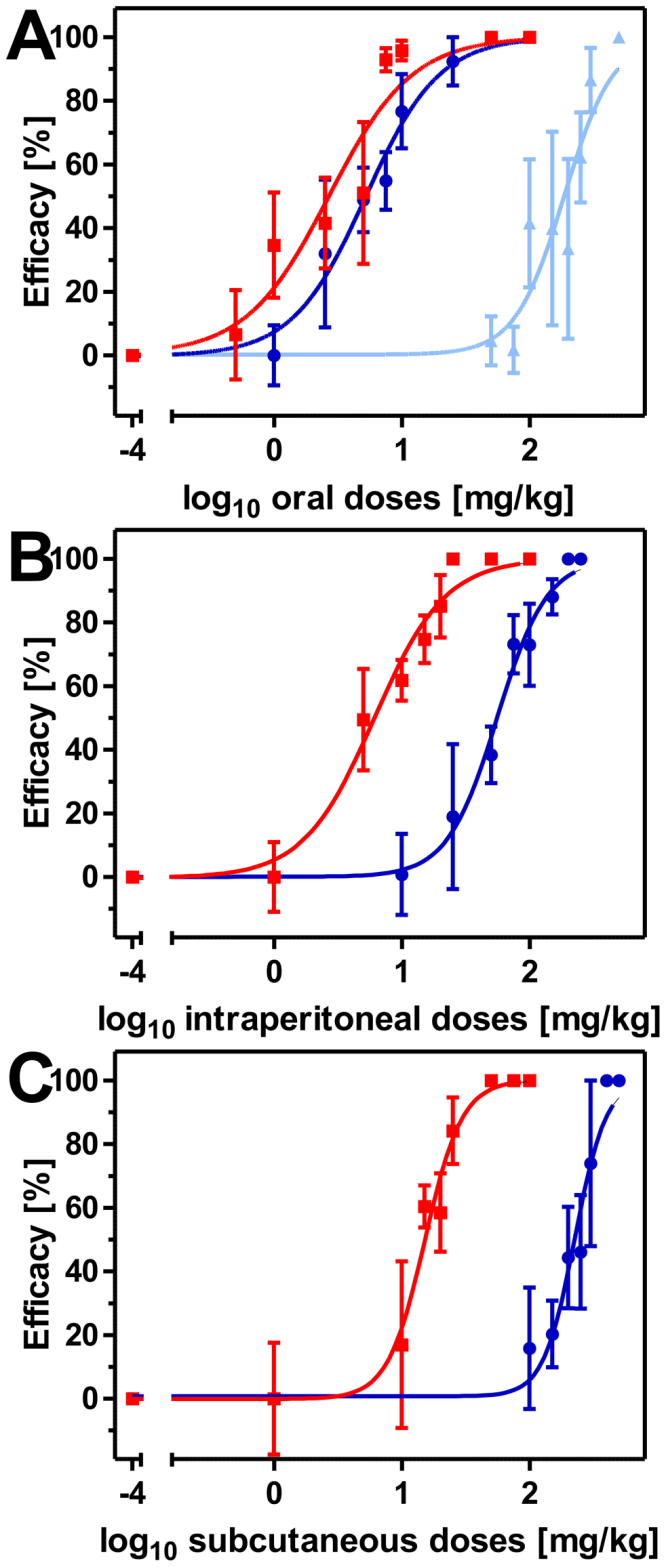
*In vivo* dose-response curves of emodepside (red) and PF1022A (blue) after oral (A), intraperitoneal (B) or subcutaneous (C) treatments against mature adults of *T. muris*. Dose-response curves show the arithmetic mean values with standard errors of the mean with a group size of five animals per drug and dose. Efficacy was calculated as relative number of recovered worms compared to the no-drug control in percentage. Dosages were log_10_ transformed and logistic regressions were calculated with top values constrained between 0 and 100%. Triangles indicate a single dose of PF1022A (light blue), circles three doses of PF1022A (dark blue) and squares three doses of emodepside (red). Efficacies were set to zero if mean of the worm counts was higher than the mean of the corresponding control group. Furthermore, the corresponding SEM values of the affected groups start from zero. The no-drug controls were set to 10^−4^ mg/kg to allow log_10_ transformation of dosages.

By comparing the three routes of administration, oral treatments diminished the worm burden at significantly lower doses than intraperitoneal or subcutaneous administrations (Figure 5A, B, C and Table 2). For emodepside, the ED_50_ values for intraperitoneal and subcutaneous treatments were approximately twofold and fivefold higher than for oral treatment (Table 2). The differences for PF1022A were even more pronounced. The ED_50_ values for intraperitoneal and subcutaneous treatments were approximately ten and 43-times higher in comparison to the ED_50_ values for the oral treatments.

By comparing the ED_50_ values of the two cyclooctadepsipeptides, the results were very diverse depending on the respective route of administration. However, the calculated ED_50_ value for PF1022A after three intraperitoneal doses was approximately nine times higher than the ED_50_ value of emodepside. A comparison of the two drugs after three subcutaneous doses resulted in an approximately 15-fold higher ED_50_ value for PF1022A. Surprisingly, the ED_50_ value of emodepside using three oral administrations was only twofold lower than that of PF1022A. Since the costs of PF1022A are much lower than those of emodepside and the difference between both drugs was only small for oral administration, a single oral dose against mature adult stages of *T. muris* was only evaluated for PF1022A.

A single oral administration of PF1022A on day 48 p.i. also resulted in dose-dependent reduction of the whipworm burden. A dose-response curve was calculated (Figure 5A) and ED_50_ and ED_95_ values with 95% CI as well as *R* ^2^ values are presented in Table 2. The ED_50_ value for PF1022A using a single oral dose was approximately 36-fold higher in comparison to the three oral administrations.

#### 3.2.3 *In vivo* efficacy of PF1022A against developmental stages of *T. muris*

**Figure 6:**
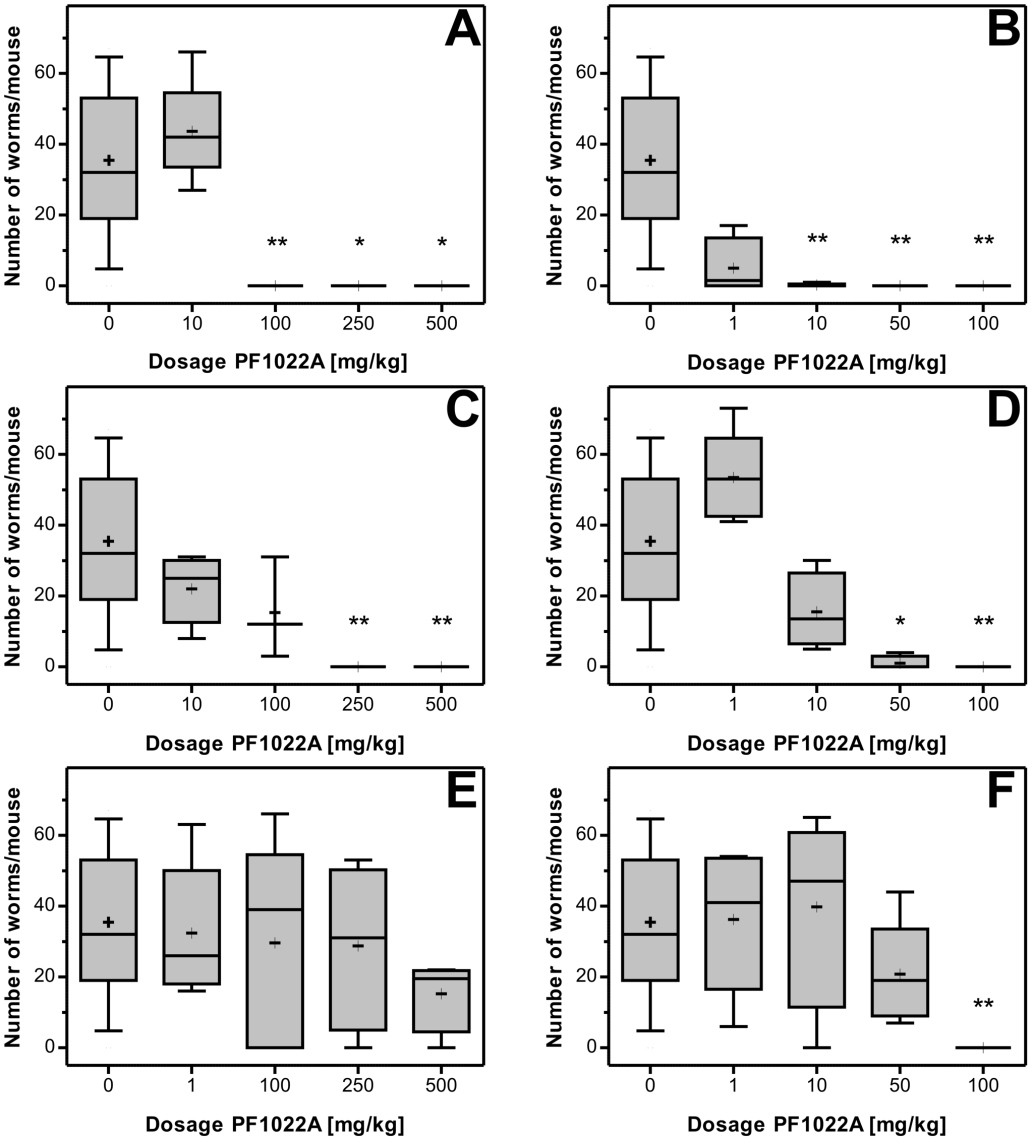
*In vivo* efficacy of PF1022A against L3, L4 and immature adults (A, B), histotropic L2 (C, D) and histotropic L1 (E, F) of *T. muris* using both, single (A, C, E) and triple (B, D, F) dose regimens. Box plots show the median numbers and quartiles of recovered adult *T. mu*ris after treatment against immature stages with wiskers representing minimal and maximal values. Group sizes were 5 mice per drug and dose. +, arithmetic mean; *, p<0.01 vs. control; **, p<0.001 vs. control.

Both single and multiple PF1022A doses on day 28 and days 26–28 resulted in dose-dependent reductions in the number of recovered worms (Figure 6A, B and Supplementary Table S3). While a single administration of 10 mg/kg did not result in any apparent effects, 100 mg/kg or higher dosages already eliminated the worm burden completely. Three oral doses of PF1022A against developmental stages on days 26–28 also resulted in nearly complete or complete cure rates starting from 10 mg/kg. Therefore, an approximately 10-fold lower dosage of PF1022A was sufficient to cure the infection with further developed larval stages and immature adult worms with three doses in comparison to a single dose.

The efficacy of single and multiple PF1022A doses on day 14 and days 12–14 respectively, targeting the histotropic L2, also resulted in dose-dependent significant reductions of the worm burden (Figure 6C, D and Supplementary Table S3). In particular, three dosages of 100 mg/kg PF1022A or a single administration of 250 mg/kg PF1022A were required for complete elimination of whipworms.

In contrast to the efficacy against L2, the effects of PF1022A against L1 were not sufficient in the single dose regimen. While three doses of 100 mg/kg PF1022A on days 1–3 p.i. were sufficient to completely cure the mice, a single dose on day 3 p.i., using even 500 mg/kg, was not able to significantly reduce worm burdens (Figure 6E, F and Supplementary Table S3).

## Discussion

The majority of human gastrointestinal nematode infections are caused by *A. lumbricoides*, *A. duodenale*, *N. americanus*, *Strongyloides. stercoralis* and *T. trichiura* [8]. Whereas available drugs are usually highly effective against *A. lumbricoides* in a single dose regimen, at least multiple dosages of those drugs are required to cure hookworm, threadworm and particularly whipworm infections. [9], [39].

In addition to the enormous impact on human medicine, the genus *Trichuris*, like *T. suis*, is also considered to be a dose-limiting nematode for most current anthelmintics in a variety of hosts of veterinary importance [13]. However, treatment options are often limited. For example, a large number of drugs (diethylcarbamazine, ivermectin, piperazine, pyrantel) registered to treat nematode infections in dogs are lacking sufficient efficacy against *T. vulpis* [12]. Among the new anthelmintics that entered the market in the recent past, especially the cyclooctadepsipeptides [21]–[23] and partially the aminophenylamidines [20], [24], [27] are active against *Trichuris* spp., whereas paraherquamide has only poor efficacy [40] and monepantel lacks efficacy [41]. For derquantel, only data describing a high efficacy of the combination with abamectin against *Trichuris ovis* have been published [42]. However, if these effects are attributed to derquantel, abamectin or only the combination of both needs to be clarified.

Persistent underdosing of *Trichuris* spp. in both humans (during the reinforced mass drug administration campaigns against lymphatic filariasis and soil-transmitted nematodes) and animals of veterinary importance may favor selection of highly resistant genotypes [9] as already described for *T. trichiura* [11]. Therefore, the urgent need for new drugs for the treatment against *Trichuris* spp., preferably in a single dose regimen, is obvious for both human and veterinary medicine.

Due to the long prepatent period of *Trichuris* spp. and the lack of efficacy of most drugs against the histotropic phase of larval forms, multiple blocks with one to three doses each are usually necessary to completely eliminate the infections [12]. Larvae of several gastrointestinal nematode species penetrate into the pits and glands of the mucosa (e.g. *Haemonchus* spp., *Ostertagia* spp., *Teladorsagia* spp.) or even penetrate and feed on individual cells (e.g. *Trichuris* spp., *Trichinella* spp.) to survive the lethargy associated with molting without losing their place in the gut [43]. These histotropic larvae are often difficult to eliminate and require higher or repeated doses when compared with luminal or mucosal stages.

In order to evaluate the effects of drugs against the histotropic stages of *T. muris*, a detailed knowledge of the time course of development within the host is required. Since data in the literature are often quite old and differ in many observations, especially regarding the number and the time course of molts (for review see [15]), the isolate used in the present study was subjected to an in-depth parasitological analysis. Furthermore, the course of infection strongly depends on the respective mouse strain [17] and *T. muris* isolate [18], making a detailed characterization even more crucial.

The parasitological data obtained here were in agreement with findings of Panesar and Croll [16]. They reported, that on day 20 p.i., all larvae were found embedded in the surface epithelium with their posterior ends extruding into the lumen of the gut. In the present study, this observation was made from day 21 onwards. In contrast to Panesar and Croll, we still found a small but significant number of histotropic stages until day 29 p.i. However, the period in which histotropic stages were exclusively present was almost the same. Interestingly, observations by Pike [37] were also in line with data shown here. They have shown, that the female/male ratio steadily develops towards more male worms and that male *T. muris* survive longer than females, which is in marked contrast to other parasitic nematode species, where females survive longer than males [37].

In the present study, no *in vivo* efficacy of the aminophenylamidine amidantel was found against patent *T. muris* infections in mice. Three oral doses of 500 mg/kg amidantel did not reduce the worm burden in comparison to the no-drug control. The efficacy of amidantel against *T. muris* was investigated previously and was also found to be only moderate [24]. Therefore, amidantel was not further evaluated in the presented study. In contrast, three consecutive oral doses with either tribendimidine or dAMD resulted in ED_50_ values of 6.5 mg/kg and 15.1 mg/kg, respectively. Complete elimination of the worm burden was achieved by three oral doses using either 25 mg/kg tribendimidine or 100 mg/kg dAMD. Oral doses of 1×400 mg [44] or 3×400 mg [26] tribendimidine have been shown to result in cure rates of 76.8% and 33.3%, respectively, against *T. trichiura* in humans. Intraperitoneal injections of the drugs, which to our knowledge were evaluated for the first time, resulted in reversed potency with ED_50_ values of 15.3 mg/kg for tribendimidine and 8.3 mg/kg for dAMD and complete elimination at dosages above 50 mg/kg in both cases. This is somewhat surprising since tribendimidine is known to rapidly disintegrate in aqueous environments releasing two molecules of dAMD [45]. Differences in release of the highly hydrophobic drugs from the used formulation (dispersion containing Cremophor EL/deionized water) are the most likely explanation for the observed phenomenon. The larger tribendimidine molecule can be suspected to diffuse more slowly into the aqueous environment. It can be assumed that release of drugs from the dispersion occurs more rapidly in the digestive track under mechanical mixing in the presence of bile salts than in the peritoneum and that passive diffusion is of minor importance in the gut. The absence of efficacy of tribendimidine and dAMD using subcutaneous administrations might also be due to the very basic formulation of the drugs. However, neither intraperitoneal nor subcutaneous administrations, using such a basic formulation, were able to significantly improve the efficacy of tribendimidine or dAMD against *T. muris* in mice.

In contrast to the aminophenylamidines, the cyclooctadepsipeptide, emodepside, has previously been shown to be completely effective against *T. vulpis* [21] and also *T. muris* [22], [23]. A single dosage of 7.16 mg/kg emodepside in the Profender spot on formulation for cats was sufficient to clear patent *T. muris* infections of mice within 48 h [22] and even treatments of mice against immature stages using 6.0 mg/kg emodespide of the same formulation on day 3, day 20 or day 35 p.i., resulted in significantly reduced worm counts (>95% efficacy) [23]. Next to the oral tablet formulation of Profender for dogs with 1 mg/kg emodepside [21], also a single dose of 0.45 mg/kg emodepside of the oral Procox suspension was sufficient to completely eliminate immature and mature *T. vulpis* from dogs [46]. However, almost all investigations on the efficacy and safety of emodepside were conducted on nematodes of veterinary importance and only few *in vitro* data on important nematodes of humans are available [47], and PF1022A has not been evaluated against *Trichuris* spp. at all. However, while no clinical signs of intolerability were found, a high degree of efficacy against a large number of helminths in a variety of hosts including *Heligmosomoides bakeri* in mice [48], *Strongyloides ratti* and *Nippostrongylus brasiliensis* in rats, *Ancylostoma caninum* in dogs, cyathostomes in horses, *Trichostrongylus colubriformis* and *Haemonchus contortus* in sheep and *Dictyocaulus viviparus* in cattle using fairly low dosages of 1–10 mg/kg PF1022A were reported [49].

There were also differences in efficacy comparing emodepside and PF1022A in the present study, but the magnitude of these differences was dependent on the route of administration. However, emodepside always performed significantly better than PF1022A using the ED_50_ value as criterion. The difference between both drugs was particularly small for the oral administration, which also performed better than the intraperitoneal and the subcutaneous route. The ED_50_ and ED_95_ values for PF1022A were only 1.9 and 1.5 fold higher than those for emodepside, respectively.

For its suitability in mass-drug-treatment programs, drugs need a high safety and production costs should be as low as possible. Due to the fact that the class of aminophenylamidines is still considered to be potentially hazardous [50] and PF1022A does have much cheaper production costs than emodepside (due to omission of semi-synthetic derivatization) [47], single dose experiments and treatments targeting developmental stages were only performed with PF1022A. In addition, no data regarding the effects of PF1022A on any stages of *Trichuris* spp. have been published previously. To examine whether PF1022A has the potential to replace the more expensive emodepside in therapy of *Trichuris* spp., it is important to determine the suitability of PF1022A as a broad-spectrum anthelmintic.

At least in the triple dose regimen, PF1022A was able to completely eliminate all developmental stages of *T. muris*. However, the required dosages inversely correlated with the time span after infection, i.e. the earlier stages had to be treated with higher dosages. Single drug administration needed 2.5 to 10-fold higher dosages to achieve complete resolution of the infections, and against L1 larvae no significant effect on worm burdens could be obtained using only a single dose. The most likely reason for this observation is the localization of the larvae. Larvae develop deep in the epithelium of the basal parts of the crypts of Lieberkühn until day 5 p.i., while they were found closer to the surface of the epithelium between days 5 and 10 p.i. On day 15 p.i., a large proportion of histotropic larvae was already found in the epithelial surface, where a higher drug concentration might be present [16].

One might think that the relatively high dosages of PF1022A required to completely eliminate developmental *T. muris* stages, especially in single dose regimens, could prevent its further development as trichuricidal drug. However, potential improvement of efficacy through optimized galenic formulations should be taken into account. The potential of cyclooctadepsipeptides for efficient treatment against *Trichuris* spp. has been shown using single oral administration of Profender tablets (Bayer Animal Health GmbH, Leverkusen, Germany). A dose rate of 1 mg/kg emodepside resulted in almost complete elimination of immature and mature stages of *T. vulpis* in dogs (>99%) [20], suggesting that optimized formulations can dramatically improve drug performance in this drug class. The formulation in Profender tablets is optimized to eliminate all relevant parasitic nematodes of dogs and optimization can be considered to improve drug efficacy. Emodepside is also the only nematocidal ingredient of Profender spot-on for cats and Procox suspension for puppies. Although using a different route of administration (dermal) Mehlhorn et al. [22] have shown that a single dosage of 7.16 mg/kg emodepside in the Profender spot on formulation for cats was sufficient to clear *T. muris* infections of mice within 48 h. In sharp contrast to that, three consecutive oral doses of 75 mg/kg emodepside using the Cremophor EL/water dispersion were required to achieve a complete elimination of patent *T. muris* infections in the present study. The more than 10-fold increase in efficacy between three doses using Cremophor/water and a single dose using the optimized Profender formulation emphasizes that every drug formulation has to be optimized for each drug and host species and that dramatic decreases in required drug dosages are possible when using an optimized formulation. In addition, optimization of formulations also decreases the risk of intoxications and the costs of treatment, in particular, if drugs can be targeted specifically towards the location of the parasite, e.g., the gut, avoiding high drug concentrations in tissues, e.g., the brain, which may be important for side effects.

In conclusion, *in vivo* treatments with relatively high doses of PF1022A resulted in complete elimination of *Trichuris muris*, including mature adult and immature adult worms as well as histotropic and further developed larval stages in a single-dose regimen. Since only non-optimized formulations were evaluated in this study, considerably lower dosages might be achievable, using formulations optimized for particular host species. Despite the fact that detailed safety and pharmacokinetic studies are still completely missing for humans, distinct effects of PF1022A against the usually dose-limiting genus *Trichuris* in the mouse model suggest that cyclooctadepsipeptides are useful candidates for development as agents against human soil-transmitted helminthoses and nematode infections of livestock animals.

## Supporting Information

Table S1 Localization of *T. muris* stages throughout prepatency.
(PDF)

Table S2 Temperature dependency of *T. muris* larval development in eggs.
(PDF)

Table S3 Descriptive statistics for each treatment group (classified by individual drugs, dose regimen and route of administration).
(PDF)
