# Supplementary material for: Efficacy of Cyclooctadepsipeptides and Aminophenylamidines against Larval, Immature and Mature Adult Stages of a Parasitologically Characterized Trichurosis Model in Mice
Source: PLoS Negl Trop Dis. 2014 Feb 20;8(2):e2698. doi: 10.1371/journal.pntd.0002698 (PMC3930511; doi:10.1371/journal.pntd.0002698)
Supplement: Table S1 — Localization of T. muris stages throughout prepatency. (PDF) [file pntd.0002698.s001.pdf]

Supplementary Table S1 Localisation of *T. muris* stages throughout prepatency

	Eggs				Free larvae/adults				Histotropic larvae				Extruding larvae			
**Day p.i.**	Duodenum	Colon	Caecum	Debris	Duodenum	Colon	Caecum	Debris	Duodenum	Colon	Caecum	Debris	Duodenum	Colon	Caecum	Debris
**1**	0, 0, 0;	0, 0, 0;	0, 0, 0;	12, 3, 0;*	0, 0, 0;	0, 0, 0;	0, 0, 0;	27, 9, 2;	0, 0, 0;	8, 5, 2;	56, 47, 10;	0, 0, 0;	0, 0, 0;	0, 0, 0;	0, 0, 0;	0, 0, 0;
**2**	0, 0, 0;	0, 0, 0;	0, 0, 0;	5, 3, 2;*	0, 0, 0;	0, 0, 0;	0, 0, 0;	17, 14, 0;	0, 0, 0;	7, 0, 0;	88, 30, 22;	0, 0, 0;	0, 0, 0;	0, 0, 0;	0, 0, 0;	0, 0, 0;
**3**	0, 0, 0;	0, 0, 0;	0, 0, 0;	4, 3, 1;*	0, 0, 0;	0, 0, 0;	0, 0, 0;	20, 19, 11;	0, 0, 0;	1, 0, 0;	13, 7, 0;	0, 0, 0;	0, 0, 0;	0, 0, 0;	0, 0, 0;	0, 0, 0;
**4**	0, 0, 0;	0, 0, 0;	0, 0, 0;	1, 0, 0;*	0, 0, 0;	0, 0, 0;	0, 0, 0;	5, 4, 0;	0, 0, 0;	0, 0, 0;	9, 5, 5;	0, 0, 0;	0, 0, 0;	0, 0, 0;	0, 0, 0;	0, 0, 0;
**5**	0, 0, 0;	0, 0, 0;	0, 0, 0;	0, 0, 0;	0, 0, 0;	0, 0, 0;	0, 0, 0;	3, 3, 1;	0, 0, 0;	0, 0, 0;	21, 12, 3;	0, 0, 0;	0, 0, 0;	0, 0, 0;	0, 0, 0;	0, 0, 0;
**6**	0, 0, 0;	0, 0, 0;	0, 0, 0;	0, 0, 0;	0, 0, 0;	0, 0, 0;	0, 0, 0;	0, 0, 0;	0, 0, 0;	1, 0, 0;	9, 4, 2;	0, 0, 0;	0, 0, 0;	0, 0, 0;	0, 0, 0;	0, 0, 0;
**7**	0, 0, 0;	0, 0, 0;	0, 0, 0;	0, 0, 0;	0, 0, 0;	0, 0, 0;	0, 0, 0;	0, 0, 0;	0, 0, 0;	2, 1, 0;	11, 7, 6;	0, 0, 0;	0, 0, 0;	0, 0, 0;	0, 0, 0;	0, 0, 0;
**8**	0, 0, 0;	0, 0, 0;	0, 0, 0;	0, 0, 0;	0, 0, 0;	0, 0, 0;	0, 0, 0;	2, 0, 0;	0, 0, 0;	0, 0, 0;	18, 13, 2;	0, 0, 0;	0, 0, 0;	0, 0, 0;	0, 0, 0;	0, 0, 0;
**9**	0, 0, 0;	0, 0, 0;	0, 0, 0;	0, 0, 0;	0, 0, 0;	0, 0, 0;	0, 0, 0;	0, 0, 0;	0, 0, 0;	3, 0, 0;	13, 12, 5;	0, 0, 0;	0, 0, 0;	0, 0, 0;	0, 0, 0;	0, 0, 0;
**10**	0, 0, 0;	0, 0, 0;	0, 0, 0;	0, 0, 0;	0, 0, 0;	0, 0, 0;	0, 0, 0;	0, 0, 0;	0, 0, 0;	5, 4, 0;	19, 16, 15;	0, 0, 0;	0, 0, 0;	0, 0, 0;	0, 0, 0;	0, 0, 0;
**11**	0, 0, 0;	0, 0, 0;	0, 0, 0;	0, 0, 0;	0, 0, 0;	0, 0, 0;	0, 0, 0;	0, 0, 0;	0, 0, 0;	0, 0, 0;	14, 12, 2;	0, 0, 0;	0, 0, 0;	0, 0, 0;	0, 0, 0;	0, 0, 0;
**12**	0, 0, 0;	0, 0, 0;	0, 0, 0;	0, 0, 0;	0, 0, 0;	0, 0, 0;	0, 0, 0;	0, 0, 0;	0, 0, 0;	0, 0, 0;	17, 10, 10;	0, 0, 0;	0, 0, 0;	0, 0, 0;	0, 0, 0;	0, 0, 0;
**13**	0, 0, 0;	0, 0, 0;	0, 0, 0;	0, 0, 0;	0, 0, 0;	0, 0, 0;	0, 0, 0;	0, 0, 0;	0, 0, 0;	0, 0, 0;	29, 26, 16;	0, 0, 0;	0, 0, 0;	0, 0, 0;	0, 0, 0;	0, 0, 0;
**14**	0, 0, 0;	0, 0, 0;	0, 0, 0;	0, 0, 0;	0, 0, 0;	0, 0, 0;	0, 0, 0;	0, 0, 0;	0, 0, 0;	1, 0, 0;	38, 24, 11;	0, 0, 0;	0, 0, 0;	0, 0, 0;	0, 0, 0;	0, 0, 0;
**15**	0, 0, 0;	0, 0, 0;	0, 0, 0;	0, 0, 0;	0, 0, 0;	0, 0, 0;	0, 0, 0;	0, 0, 0;	0, 0, 0;	0, 0, 0;	26, 20, 18;	0, 0, 0;	0, 0, 0;	0, 0, 0;	0, 0, 0;	0, 0, 0;
**16**	0, 0, 0;	0, 0, 0;	0, 0, 0;	0, 0, 0;	0, 0, 0;	0, 0, 0;	0, 0, 0;	0, 0, 0;	0, 0, 0;	8, 2, 0;	29, 25, 10;	0, 0, 0;	0, 0, 0;	0, 0, 0;	0, 0, 0;	0, 0, 0;
**17**	0, 0, 0;	0, 0, 0;	0, 0, 0;	0, 0, 0;	0, 0, 0;	0, 0, 0;	0, 0, 0;	0, 0, 0;	0, 0, 0;	2, 0, 0;	24, 15, 14;	0, 0, 0;	0, 0, 0;	0, 0, 0;	0, 0, 0;	0, 0, 0;
**18**	0, 0, 0;	0, 0, 0;	0, 0, 0;	0, 0, 0;	0, 0, 0;	0, 0, 0;	0, 0, 0;	0, 0, 0;	0, 0, 0;	4, 4, 1;	43, 31, 28;	0, 0, 0;	0, 0, 0;	0, 0, 0;	0, 0, 0;	0, 0, 0;
**19**	0, 0, 0;	0, 0, 0;	0, 0, 0;	0, 0, 0;	0, 0, 0;	0, 0, 0;	0, 0, 0;	0, 0, 0;	0, 0, 0;	11, 5, 0;	59, 39, 37;	0, 0, 0;	0, 0, 0;	0, 0, 0;	0, 0, 0;	0, 0, 0;
**20**	0, 0, 0;	0, 0, 0;	0, 0, 0;	0, 0, 0;	0, 0, 0;	0, 0, 0;	0, 0, 0;	0, 0, 0;	0, 0, 0;	3, 1, 0;	47, 41, 9;	0, 0, 0;	0, 0, 0;	0, 0, 0;	0, 0, 0;	0, 0, 0;
**21**	0, 0, 0;	0, 0, 0;	0, 0, 0;	0, 0, 0;	0, 0, 0;	0, 0, 0;	0, 0, 0;	0, 0, 0;	0, 0, 0;	0, 0, 0;	33, 30, 21;	0, 0, 0;	0, 0, 0;	0, 0, 0;	8, 2, 0;	0, 0, 0;
**22**	0, 0, 0;	0, 0, 0;	0, 0, 0;	0, 0, 0;	0, 0, 0;	0, 0, 0;	0, 0, 0;	0, 0, 0;	0, 0, 0;	0, 0, 0;	12, 3, 3;	0, 0, 0;	0, 0, 0;	5, 2, 0;	17, 13, 3;	0, 0, 0;
**23**	0, 0, 0;	0, 0, 0;	0, 0, 0;	0, 0, 0;	0, 0, 0;	0, 0, 0;	0, 0, 0;	0, 0, 0;	0, 0, 0;	0, 0, 0;	13, 8, 7;	0, 0, 0;	0, 0, 0;	1, 1, 0;	19, 12, 6;	0, 0, 0;
**24**	0, 0, 0;	0, 0, 0;	0, 0, 0;	0, 0, 0;	0, 0, 0;	0, 0, 0;	0, 0, 0;	0, 0, 0;	0, 0, 0;	0, 0, 0;	5, 0, 0;	0, 0, 0;	0, 0, 0;	11, 0, 0;	54, 27, 4;	0, 0, 0;
**25**	0, 0, 0;	0, 0, 0;	0, 0, 0;	0, 0, 0;	0, 0, 0;	0, 0, 0;	0, 0, 0;	0, 0, 0;	0, 0, 0;	0, 0, 0;	5, 4, 0;	0, 0, 0;	0, 0, 0;	8, 0, 0;	79, 40, 23;	0, 0, 0;
**26**	0, 0, 0;	0, 0, 0;	0, 0, 0;	0, 0, 0;	0, 0, 0;	0, 0, 0;	0, 0, 0;	0, 0, 0;	0, 0, 0;	2, 0, 0;	1, 1, 0;	0, 0, 0;	0, 0, 0;	9, 9, 1;	35, 29, 6;	0, 0, 0;
**27**	0, 0, 0;	0, 0, 0;	0, 0, 0;	0, 0, 0;	0, 0, 0;	0, 0, 0;	0, 0, 0;	9, 0, 0;	0, 0, 0;	0, 0, 0;	2, 1, 0;	0, 0, 0;	0, 0, 0;	25, 3, 2;	62, 10, 8;	0, 0, 0;
**28**	0, 0, 0;	0, 0, 0;	0, 0, 0;	0, 0, 0;	0, 0, 0;	0, 0, 0;	0, 0, 0;	23, 0, 0;	0, 0, 0;	1, 0, 0;	0, 0, 0;	0, 0, 0;	0, 0, 0;	17, 11, 0;	36, 19, 1;	0, 0, 0;
**29**	0, 0, 0;	0, 0, 0;	0, 0, 0;	0, 0, 0;	0, 0, 0;	0, 0, 0;	0, 0, 0;	3, 2, 0;	0, 0, 0;	0, 0, 0;	3, 0, 0;	0, 0, 0;	0, 0, 0;	22, 20, 5;	98, 55, 5;	0, 0, 0;
**30**	0, 0, 0;	0, 0, 0;	0, 0, 0;	82, 0, 0;**	0, 0, 0;	0, 0, 0;	0, 0, 0;	6, 0, 0;	0, 0, 0;	0, 0, 0;	0, 0, 0;	0, 0, 0;	0, 0, 0;	13, 10, 0;	71, 38, 27;	0, 0, 0;
**31**	0, 0, 0;	0, 0, 0;	0, 0, 0;	327, 0, 0;**	0, 0, 0;	0, 0, 0;	0, 0, 0;	5, 1, 1;	0, 0, 0;	0, 0, 0;	0, 0, 0;	0, 0, 0;	0, 0, 0;	7, 5, 0;	54, 31, 23;	0, 0, 0;

Table shows localization of *T. muris* stages within their murine host in the course of prepatent period. Stages are subdivided into (i) eggs, (ii) larvae found in the intestinal contents (free larvae), (iii) larvae completely penetrated into the epithelium of duodenum, colon or caecum (histotropic larvae) and (iv) larvae extruding their posterior ends into the lumen of the guts (extruding larvae). Absolute numbers of stage counts per region are given for each mouse individually.*, fully embryonated eggs; **, unembryonated eggs;
