# Supplementary material for: Efficacy of Cyclooctadepsipeptides and Aminophenylamidines against Larval, Immature and Mature Adult Stages of a Parasitologically Characterized Trichurosis Model in Mice
Source: PLoS Negl Trop Dis. 2014 Feb 20;8(2):e2698. doi: 10.1371/journal.pntd.0002698 (PMC3930511; doi:10.1371/journal.pntd.0002698)
Supplement: Table S2 — Temperature dependency of T. muris larval development in eggs. (PDF) [file pntd.0002698.s002.pdf]

Supplementary Table S2 Temperature dependency of *T. muris* larval development in eggs.

	Unsegmented (in %)				Partially segmented (in %)				Fully developed (in %)				Degenerated (in %)			
**DPP**	**4°C**	**19°C**	**27°C**	**37°C**	**4°C**	**19°C**	**27°C**	**37°C**	**4°C**	**19°C**	**27°C**	**37°C**	**4°C**	**19°C**	**27°C**	**37°C**
**0**	100	100	100	100	0	0	0	0	0	0	0	0	0	0	0	0
	100	100	100	100	0	0	0	0	0	0	0	0	0	0	0	0
	100	100	100	100	0	0	0	0	0	0	0	0	0	0	0	0
	100	100	100	100	0	0	0	0	0	0	0	0	0	0	0	0
	100	100	100	100	0	0	0	0	0	0	0	0	0	0	0	0
	100	100	100	100	0	0	0	0	0	0	0	0	0	0	0	0
**7**	100	100	100	100	0	0	0	0	0	0	0	0	0	0	0	0
	100	100	100	100	0	0	0	0	0	0	0	0	0	0	0	0
	100	100	100	100	0	0	0	0	0	0	0	0	0	0	0	0
	100	100	100	100	0	0	0	0	0	0	0	0	0	0	0	0
	100	100	100	100	0	0	0	0	0	0	0	0	0	0	0	0
	100	100	100	100	0	0	0	0	0	0	0	0	0	0	0	0
**14**	100	100	100	100	0	0	0	0	0	0	0	0	0	0	0	0
	100	100	100	100	0	0	0	0	0	0	0	0	0	0	0	0
	100	100	100	100	0	0	0	0	0	0	0	0	0	0	0	0
	100	100	100	96.0	0	0	0	4.0	0	0	0	0	0	0	0	0
	100	100	100	89.5	0	0	0	10.5	0	0	0	0	0	0	0	0
	100	100	94.7	75.0	0	0	5.3	25.0	0	0	0	0	0	0	0	0
**21**	100	100	100	0	0	0	0	100	0	0	0	0	0	0	0	0
	100	100	95.0	10.5	0	0	5.0	89.5	0	0	0	0	0	0	0	0
	100	100	88.9	11.7	0	0	11.1	88.3	0	0	0	0	0	0	0	0
	100	100	86.9	15.8	0	0	13.1	84.2	0	0	0	0	0	0	0	0
	100	100	76.2	22.8	0	0	23.8	77.2	0	0	0	0	0	0	0	0
	100	86.4	68.4	33.3	0	13.7	31.6	66.7	0	0	0	0	0	0	0	0
**28**	100	100	73.7	0	0	0	26.3	100	0	0	0	0	0	0	0	0
	100	100	68.4	0	0	0	31.6	100	0	0	0	0	0	0	0	0
	100	100	61.9	0	0	0	38.1	100	0	0	0	0	0	0	0	0
	100	69.6	48.0	0	0	30.4	52.0	61.9	0	0	0	0	0	0	0	38.9
	100	61.1	40.0	0	0	38.9	60.0	57.1	0	0	0	0	0	0	0	42.9
	100	55.2	36.8	0	0	44.8	64.8	42.9	0	0	0	9.5	0	0	0	47.6
**35**	100	81.1	0	0	0	18.9	100	5.0	0	0	0	20.0	0	0	0	75.0
	100	76.5	0	0	0	23.5	100	3.7	0	0	0	25.9	0	0	0	70.4

	100	73.3	0	0	0	26.7	95.7	0	0	0	0	44.0	0	0	4.3	66.0
	100	26.9	7.4	0	0	73.1	92.6	0	0	0	0	82.1	0	0	0	17.9
	100	26.7	5.3	0	0	73.3	94.7	0	0	0	0	71.4	0	0	0	28.6
	100	5.9	4.4	0	0	94.1	95.6	0	0	0	0	87.5	0	0	0	12.5
**42**	100	78.9	0	0	0	21.1	73.3	0	0	0	26.7	73.3	0	0	0	26.7
	100	78.6	0	0	0	21.4	72.7	0	0	0	27.3	76.2	0	0	0	23.8
	100	80.0	0	0	0	20.0	70.0	0	0	0	30.0	72.7	0	0	0	27.3
	100	0	0	0	0	100	52.9	0	0	0	47.1	35.7	0	0	0	64.3
	100	0	0	0	0	100	41.2	0	0	0	58.8	35.3	0	0	0	64.7
	100	0	0	0	0	100	35.7	0	0	0	64.3	12.5	0	0	0	87.5
**49**	100	54.2	0		0	45.8	42.9		0	0	57.1		0	0	0	
	100	41.4	0		0	58.6	36.4		0	0	63.6		0	0	0	
	100	38.9	0		0	61.1	35.0		0	0	65.0		0	0	0	
	100	0	0		0	100	14.3		0	0	85.7		0	0	0	
	100	0	0		0	100	11.8		0	0	88.2		0	0	0	
	100	0	0		0	100	10.0		0	0	90.0		0	0	0	
**56**	100	0	0		0	100	0		0	0	100		0	0	0	
	100	0	0		0	100	0		0	0	100		0	0	0	
	100	0	0		0	100	0		0	0	100		0	0	0	
	100	42.1	0		0	57.9	0		0	0	100		0	0	0	
	100	40.9	0		0	59.1	0		0	0	96.6		0	0	4.4	
	100	33.3	0		0	66.7	0		0	0	94.7		0	0	5.3	
**63**	100	0			0	100			0	0			0	0		
	100	0			0	100			0	0			0	0		
	100	0			0	100			0	0			0	0		
	100	0			0	100			0	0			0	0		
	100	0			0	100			0	0			0	0		
	100	10.5			0	89.5			0	0			0	0		
**70**	100	0			0	100			0	0			0	0		
	100	0			0	100			0	0			0	0		
	100	0			0	100			0	0			0	0		
	100	0			0	100			0	0			0	0		
	100	4.5			0	95.5			0	0			0	0		
	100	5.9			0	94.1			0	0			0	0		

DPI: Days post purification.
