# Supplementary material for: Efficacy of Cyclooctadepsipeptides and Aminophenylamidines against Larval, Immature and Mature Adult Stages of a Parasitologically Characterized Trichurosis Model in Mice
Source: PLoS Negl Trop Dis. 2014 Feb 20;8(2):e2698. doi: 10.1371/journal.pntd.0002698 (PMC3930511; doi:10.1371/journal.pntd.0002698)
Supplement: Table S3 — Descriptive statistics for each treatment group (classified by individual drugs, dose regimen and route of administration). (PDF) [file pntd.0002698.s003.pdf]

Supplementary Table S3: Descriptive statistics for each treatment group (classified by individual drugs, dose regimen and route of administration)

dAMD, three oral doses

	Control	1.0 mg/kg	5.0 mg/kg	10 mg/kg	25 mg/kg	50 mg/kg	100 mg/kg
**Mean**	32.97	46.80	56.00	23.60	11.20	2.33	0
**SD**	13.66	12.03	16.93	15.95	9.83	3.83	0
**N**	89	5	5	5	5	5	5

dAMD, three intraperitoneal doses

	Control	5.0 mg/kg	10 mg/kg	25 mg/kg	50 mg/kg	100 mg/kg
**Mean**	32.97	36.20	9.40	3.40	0	0
**SD**	13.66	14.45	5.98	5.41	0	0
**N**	89	5	5	5	5	5

Tribendimidine, three oral doses

	Control	1.0 mg/kg	5.0 mg/kg	10 mg/kg	25 mg/kg	50 mg/kg	100 mg/kg
**Mean**	32.97	59.20	35.00	5.80	0	0	0
**SD**	13.66	22.00	9.03	5.72	0	0	0
**N**	89	5	5	5	5	5	5

Tribendimidine, three intraperitoneal doses

	Control	5.0 mg/kg	10 mg/kg	25 mg/kg	50 mg/kg	100 mg/kg
**Mean**	32.97	36.80	27.60	8.80	0	0
**SD**	13.66	14.03	6.62	7.85	0	0
**N**	89	5	5	5	5	5

PF1022A, three oral doses

	Control	1.0 mg/kg	2.5 mg/kg	5.0 mg/kg	7.5 mg/kg	10 mg/kg	25 mg/kg	50 mg/kg	100 mg/kg
**Mean**	32.97	36.60	26.80	13.60	17.80	6.20	3.00	0	0
**SD**	13.66	5.59	20.46	6.03	7.98	6.94	6.71	0	0
**N**	89	5	5	5	5	5	5	5	5

PF1022A, three intraperitoneal doses

	Control	10 mg/kg	25 mg/kg	50 mg/kg	75 mg/kg	100 mg/kg	150 mg/kg	200 mg/kg	250 mg/kg
**Mean**	32.97	48.00	39.20	29.80	13.00	5.20	5.80	0	0
**SD**	13.66	13.75	24.61	9.63	9.92	5.54	6.02	0	0
**N**	89	5	5	5	5	5	5	5	5

PF1022A, three subcutaneous doses

	Control	50 mg/kg	100 mg/kg	150 mg/kg	200 mg/kg	250 mg/kg	300 mg/kg	400 mg/kg	500 mg/kg
**Mean**	32.97	32.60	16.20	21.60	15.80	15.30	7.40	0	0
**SD**	13.66	7.50	8.23	7.27	10.13	11.32	16.55	0	0
**N**	89	5	5	5	5	5	5	5	5

PF1022A, one oral dose

	Control	50 mg/kg	75 mg/kg	100 mg/kg	150 mg/kg	200 mg/kg	250 mg/kg	300 mg/kg	500 mg/kg
**Mean**	32.97	33.20	33.40	11.00	16.00	12.80	12.40	2.60	0
**SD**	13.66	5.97	5.50	8.46	18,10	12.15	10.36	4.34	0
**N**	89	5	5	5	5	5	5	5	5

Emodepside, three oral doses

	Control	0.5 mg/kg	1.0 mg/kg	2.5 mg/kg	5.0 mg/kg	7.5 mg/kg	10 mg/kg	50 mg/kg	100 mg/kg
**Mean**	32.97	28.80	12.29	18.00	9.20	2o20	0.80	0	0
**SD**	13.66	9.68	6.94	9.82	9.36	2,49	1.30	0	0
**N**	89	5	5	5	5	5	5	5	5

Emodepside, three intraperitoneal doses

	Control	1.0 mg/kg	5.0 mg/kg	10 mg/kg	15 mg/kg	20 mg/kg	25 mg/kg	50 mg/kg	100 mg/kg
**Mean**	32.97	37.00	17.60	13.00	8.80	5.20	0	0	0
**SD**	13.66	8.34	12.40	4.85	5.85	7.66	0	0	0
**N**	89	5	5	5	5	5	5	5	5

Emodepside, three subcutaneous doses

	Control	1.0 mg/kg	10 mg/kg	15 mg/kg	20 mg/kg	25 mg/kg	50 mg/kg	75 mg/kg	100 mg/kg
**Mean**	32.97	37.00	17.60	13.00	8.80	5.20	0	0	0
**SD**	13.66	8.34	12.40	4.85	5,85	7.66	0	0	0
**N**	89	5	5	5	5	5	5	5	5
